# 5-HT_2A_ Gene Variants Moderate the Association between PTSD and Reduced Default Mode Network Connectivity

**DOI:** 10.3389/fnins.2016.00299

**Published:** 2016-06-28

**Authors:** Mark W. Miller, Emily Sperbeck, Meghan E. Robinson, Naomi Sadeh, Erika J. Wolf, Jasmeet P. Hayes, Mark Logue, Steven A. Schichman, Angie Stone, William Milberg, Regina McGlinchey

**Affiliations:** ^1^Behavioral Science Division, National Center for PTSD, VA Boston Healthcare SystemBoston, MA, USA; ^2^Department of Psychiatry, Boston University School of MedicineBoston, MA, USA; ^3^Neuroimaging Research for Veterans Center, VA Boston Healthcare SystemBoston, MA, USA; ^4^Geriatric Research Educational and Clinical Center and Translational Research Center for TBI and Stress Disorders, VA Boston Healthcare SystemBoston, MA, USA; ^5^Department of Neurology, Boston University School of MedicineBoston, MA, USA; ^6^Biomedical Genetics, Boston University School of MedicineBoston, MA, USA; ^7^Department of Biostatistics, Boston University School of Public HealthBoston, MA, USA; ^8^Pharmacogenomics Analysis Laboratory, Research Service, Central Arkansas Veterans Healthcare SystemLittle Rock, AR, USA; ^9^Department of Psychiatry, Harvard Medical SchoolBoston, MA, USA

**Keywords:** posttraumatic stress disorder, default mode network, functional connectivity, serotonin receptor, *HTR1B*, *HTR2A*

## Abstract

The default mode network (DMN) has been used to study disruptions of functional connectivity in a wide variety of psychiatric and neurological conditions, including posttraumatic stress disorder (PTSD). Studies indicate that the serotonin system exerts a modulatory influence on DMN connectivity; however, no prior study has examined associations between serotonin receptor gene variants and DMN connectivity in either clinical or healthy samples. We examined serotonin receptor single nucleotide polymorphisms (SNPs), PTSD, and their interactions for association with DMN connectivity in 134 White non-Hispanic veterans. We began by analyzing candidate SNPs identified in prior meta-analyses of relevant psychiatric traits and found that rs7997012 (an *HTR2A* SNP), implicated previously in anti-depressant medication response in the Sequenced Treatment Alternatives for Depression study (STAR^*^D; McMahon et al., 2006), interacted with PTSD to predict reduced connectivity between the posterior cingulate cortex (PCC) and the right medial prefrontal cortex and right middle temporal gyrus (MTG). rs130058 (*HTR1B*) was associated with connectivity between the PCC and right angular gyrus. We then expanded our analysis to 99 *HTR1B* and *HTR2A* SNPs and found two *HTR2A* SNPs (rs977003 and rs7322347) that significantly moderated the association between PTSD severity and the PCC-right MTG component of the DMN after correcting for multiple testing. Finally, to obtain a more precise localization of the most significant SNP × PTSD interaction, we performed a whole cortex vertex-wise analysis of the rs977003 effect. This analysis revealed the locus of the pre-frontal effect to be in portions of the superior frontal gyrus, while the temporal lobe effect was centered in the middle and inferior temporal gyri. These findings point to the influence of *HTR2A* variants on DMN connectivity and advance knowledge of the role of 5-HT_2A_ receptors in the neurobiology of PTSD.

## Introduction

The default mode network (DMN) is a coherent network of spontaneous neural activity that is enhanced during tasks involving internal mentation such as autobiographical memory, self-referential thinking and mind-wandering (Mason et al., 2007; Spreng et al., 2009; Andrews-Hanna, 2012). Conversely, it is suppressed during externally-oriented and attention-demanding cognition and goal-directed behavior (Gusnard and Raichle, 2001; Buckner et al., 2008). The DMN is typically modeled by examining the time-course of blood oxygenation level-dependent (BOLD) signal covariation between the posterior cingulate cortex (PCC) and the remainder of the cortex and identifying regions showing the strongest correlations with the PCC. The PCC and anterior medial prefrontal cortex are the primary hubs of the DMN. Other core regions are organized along the anterior and posterior midline (Buckner et al., 2008) with functionally distinct subdivisions including the dorsal medial prefrontal cortex and ventral medial prefrontal cortex, medial temporal lobe, and lateral parietal cortex (Andrews-Hanna et al., 2010). The DMN is reliably measured, well-characterized, and has garnered considerable interest as a construct for advancing understanding of relationships between disruptions of intrinsic connectivity networks and a wide variety of psychiatric and neurological conditions (Broyd et al., 2009; Raichle, 2015). Twin studies have shown the DMN to be under considerable genetic control, with some heritability estimates exceeding 40% (Glahn et al., 2010; Korgaonkar et al., 2014), which raises the possibility that it could serve as an endophenotype for brain disorders with a genetic component, including PTSD.

Serotonergic pathways from the midbrain raphe nucleus project directly onto regions of the DMN including the cingulate cortex (Moore et al., 1978), and recent studies suggest that connectivity of the network may be modulated by activity of this neurotransmitter system. Hahn et al. (2012) measured 5-HT_1A_ receptor binding using positron emission tomography and examined associations between binding potential in regions implicated in the DMN and resting state activity of the same areas recorded using functional magnetic resonance imaging (fMRI). Analyses revealed significant negative associations between levels of 5-HT_1A_ heteroreceptors and BOLD signal in the dorsal medial prefrontal cortex (mPFC) and PCC suggesting a modulatory influence of these receptors on DMN connectivity in these regions. Another study found that peripheral platelet serotonin uptake velocity (a model of neuronal transport that measures the efficiency with which serotonin is transported across the cell membrane by the serotonin transporter) was negatively correlated with DMN activity in the mPFC (Scharinger et al., 2014). In addition, administration of a 5-HT_2A_/5-HT_2C_ receptor agonist has been shown to decrease coupling between the mPFC and the PCC (Carhart-Harris et al., 2012), and serotonin reuptake inhibitors (SSRI) reduce pairwise connectivity between several regions of the DMN in healthy subjects (van de Ven et al., 2013; Klaassens et al., 2015). Together, these findings point to a possible modulatory influence of serotonin signaling on DMN activity.

Based on evidence for genetic and serotonergic modulation of the DMN along with extensive prior research implicating the serotonin system in the neurobiology of PTSD (Krystal and Neumeister, 2009), we undertook an examination of possible associations between serotonin receptor gene polymorphisms and DMN connectivity in a sample of military veterans with a high prevalence of current PTSD. Serotonin-related polymorphisms have been linked previously to risk for psychiatric illness and relevant psychiatric traits (see Table 1 for serotonin receptor gene meta-analyses and references (Munafò et al., 2008; Karg et al., 2011) for serotonin transporter/*5-HTTLPR* meta-analyses), but only two previous studies have examined the association between the genetic variation within this system and DMN connectivity. Wiggins et al. (2012) examined the influence of *5-HTTLPR* variants on DMN connectivity in a sample of 39 healthy children and adolescents and found that individuals homozygous for the S allele [linked in other research to lower expression (Heils et al., 1996) and greater amygdala reactivity (Hariri et al., 2005; Pezawas et al., 2005)] showed weaker connectivity in the superior medial frontal cortex. The same group reported a similar pattern among healthy control subjects in a subsequent study^1^ (Wiggins et al., 2013). To our knowledge, however, no prior study has examined serotonin receptor polymorphisms for possible association with DMN connectivity in either healthy or psychiatric samples. We began our research into this topic by reviewing published quantitative meta-analyses of studies that have examined associations between serotonin receptor gene variants and relevant psychiatric traits. From this, we identified 7 candidate SNPs in the *HTR1A, HTR1B, HTR2A*, and *HTR3B* genes showing evidence of replicable association with phenotypes ranging from anti-depressant treatment response to mood and anxiety disorders, attention deficit hyperactivity, and schizophrenia (see Table 1). We then examined these SNPs for association with DMN connectivity and PTSD.

**Table 1:** **Serotonin receptor gene candidate SNPs and the meta-analyses supporting their associations**.

**Gene**	**SNP**	**AKA^a^**	**Proxy SNP^b^**	**Supporting meta-analysis**
*HTR1A*	rs6295	1019C/G	rs878567	Depression (Kishi et al., 2009, 2013), Bipolar Disorder (Kishi et al., 2011)
*HTR1B*	rs6296	861G/C	rs6298	ADHD (Gizer et al., 2009)
	rs130058	–161A/T		Substance Abuse (Cao et al., 2013)
	rs11568817	–261T/G		Substance Abuse (Cao et al., 2013)
*HTR2A*	rs7997012			Anti-depressant response (Niitsu et al., 2013; Lin et al., 2014)
	rs6313	102T>C	rs6311	Anti-depressant response (Kato and Serretti, 2010; Niitsu et al., 2013; Lin et al., 2014); OCD (Taylor, 2013), Schizophrenia (Gu et al., 2013; Gatt et al., 2015), Substance Abuse (Cao et al., 2014), Panic Disorder (Howe et al., 2016)
*HTR3B*	rs1176744	p.Y129S		Bipolar Disorder (Hammer et al., 2012)

a AKA = “also known as.”

b *We use the term “proxy SNP” here refer to SNPs in high LD with the candidate SNP*.

Prior neuroimaging studies have found PTSD to be associated with reduced resting state and task-based activation of regions implicated in the default network such as the medial prefrontal cortex (for meta-analyses, see Hayes et al., 2012; Sartory et al., 2013; Koch et al., 2016), leading investigators to hypothesize that some of the hallmark symptoms of PTSD (e.g., re-experiencing and hypervigilance) may be indicative of a failure in resting neural regulatory mechanisms (Sripada et al., 2012; Lanius et al., 2015; Reuveni et al., 2016). However, extant studies of the DMN in PTSD have been limited by small samples and there have been mixed results. Several studies reported negative correlations between PTSD and DMN connectivity (Bluhm et al., 2009 [*N* = 32]; Sripada et al., 2012 [*N* = 30]; and Zhou et al., 2012 [*N* = 15]), while others have found positive associations (Lanius et al., 2010; *N* = 11) and Reuveni et al., 2016; *N* = 20 or a mixed pattern (Qin et al., 2012; *N* = 29). Given the foregoing evidence for the modulatory influence of genes and serotonin on the DMN, we wondered if some of the variability in results observed across studies might relate to previously unaccounted for genetic variation in serotonin signaling and hypothesized that serotonin receptor gene SNPs would moderate associations between PTSD and DMN connectivity. We recently examined the effects of blast exposure and mild traumatic brain injury (mTBI) on DMN connectivity using the cohort on which this report was based (*N* = 134) and found blast exposure to be associated with reduced connectivity in the bilateral primary somatosensory and motor cortices; however, associations between PTSD and DMN connectivity were not thoroughly examined (Robinson et al., 2015). The aim of this study, therefore, was to revisit the relationship between PTSD and DMN activity using an analysis designed to identify genetic variants that influence DMN connectivity directly and/or modify its association with PTSD.

## Materials and methods

### Participants

Participants were United States veterans of the conflicts in Iraq and/or Afghanistan consecutively enrolled in the Translational Research Center for TBI and Stress Disorders at VA Boston Healthcare System. Exclusion criteria included history of seizures unrelated to head injury, neurological illness, current psychotic or bipolar disorder, severe depression or anxiety, active homicidal and/or suicidal ideation with intent, cognitive disorder due to general medical condition other than traumatic brain injury (TBI), and unstable psychological diagnosis that would interfere with accurate data collection. Additional MRI exclusion criteria included pregnancy and having a metal implant, shrapnel, aneurysm clip, or pacemaker. Of those who were genotyped and had DMN data, 154 were of self-reported White non-Hispanic (WNH) ancestry. Ancestry was confirmed via genetic principal components (PC) analysis of genome-wide SNP data (see Supplementary Material) and eight subjects were dropped because they had PC values > 6 standard deviations away from the mean for WNH subjects. An additional 11 subjects were omitted from analysis due to excessive motion during the restating state scan. Of the remaining 135 participants, 124 (91.9%) were male and their mean age was 31.39 (*SD* = 7.97, Range: 19–57 years). Most veterans had served in the Army (63.7%), but the Marines (23%), Air Force (8.9%), Navy (3.7%), and Coast Guard (0.7%) were also represented. Additional clinical characteristics can be found in Robinson et al. (2015) and Table 2.

**Table 2:** **Sample descriptives for male veterans by current PTSD diagnosis**.

	**PTSD**	**No PTSD**	***t***	***p***
	**Mean (SD)**	**Mean (SD)**		
N (%)	65 (52.8%)	58 (47.2%)		
Age	30.3 (6.4)	32.4 (9.5)	1.417	0.159
CAPS Severity	65.6 (17.3)	18.1 (13.5)	16.837	<0.001
Blast Exposure	1.82 (4.1)	1.00 (3.6)	1.152	0.252
Combat Exposure	20.4 (12.9)	10.9 (8.6)	4.606	<0.001
MDD Diagnosis	0.22 (0.4)	0 (0)	3.958	<0.001
Antidepressant Medication	0.28 (0.5)	0.05 (0.2)	3.444	0.001

*CAPS, Clinician Administered PTSD Scale current severity score; Blast exposure, number of close range blast exposures (<10 m); Combat Exposure, DRRI Combat Experience total score; MDD diagnosis, current diagnosis of major depressive disorder, single episode or recurring*.

### Procedures and measures

Participants underwent a series of clinical interviews, MRI scans, and had blood samples drawn for genotyping. The VA Boston Healthcare System and Boston University Medical Campus Institutional Review Boards approved the study and participants gave written informed consent.

#### MRI data acquisition, processing, and analysis

**Figure 1:**
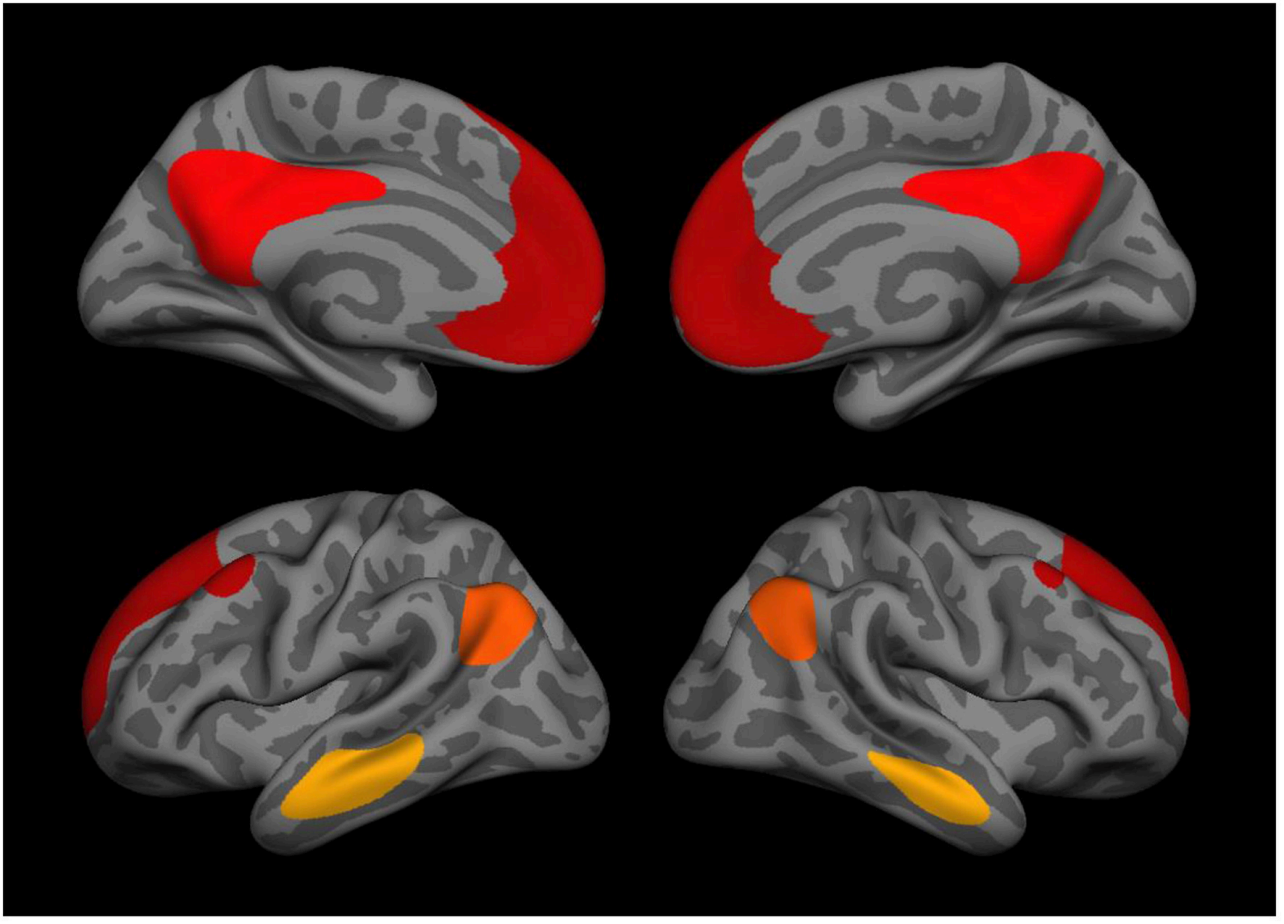
**Medial (top) and lateral (bottom) views of the 8 DMN regions used in the genetic association analysis including the isthmus of the cingulate, angular gyrus, middle temporal gyrus, and medial prefrontal cortex (each bilateral)**.

Neuroimaging data were acquired on a Siemens 3T TIM Trio system with a 12-radiofrequency channels head coil. Details about the scan acquisition and processing methods are available in Robinson et al. (2015). In brief, two anatomical scans were acquired for surface reconstruction, functional connectivity seed placement, and inter-participant registration. Resting state functional data were acquired in two 2 runs (6 min each) with participants instructed to keep their eyes open and stay awake prior to each run. Intervals with excessive motion were excluded, data were sampled to and smoothed on the surface, and each brain was warped to a surface-based template using FreeSurfer (Fischl et al., 2002). Seed regions were derived from surface-based parcellations of the cortex (Fischl, 2012). The bilateral superior third of the isthmus of the cingulate (posterior portion) as defined within each participant's native space was used as a seed region. Group-level surface-based connectivity maps were assessed in the cohort (without restriction for ancestry) and clustered at a vertex-wise threshold of *p* < 10^−20^, yielding four regions of interest (ROIs) from each hemisphere. These were: bilateral isthmus of the cingulate, angular gyrus, middle temporal gyrus (MTG), and medial pre-frontal cortex (mPFC; see Figure 1). The matrix of correlations between each component is listed in Table S1. Finally, we performed a whole cortex vertex-wise analysis of the most significant PTSD × SNP interaction to obtain a more precise localization of the effect using the same seed regions along with the covariates (age, sex, and the first two genetic PCs) as described in the data analysis section below. Correction for multiple comparisons was accomplished through Monte Carlo simulation using a vertex-wise threshold of *p* = 0.05.

#### Genotyping

Genetic association analyses were conducted using a subset of ancestrally homogenous participants (*n* = 135 White, non-Hispanic participants) who also had DMN and clinical data available for analysis. DNA was isolated from blood on a Qiagen AutoPure instrument using Qiagen reagents and samples were normalized using PicoGreen assays (Invitrogen). Genotyping was based on the Illumina HumanOmni2.5-8 microarray and scanned using an Illumina iScan System (Illumina, San Diego, CA). Of the 7 candidate SNPs implicated in meta-analyses of relevant psychiatric traits, 4 were on the Omni2.5 chip (rs130058, rs6313, rs7997012, rs1176744) and the other three (rs6295, rs6296, rs11568817) were imputed from 1000 Genomes (1000 Genomes Project Consortium, 2012). We performed follow-up analyses in genes with significant associations (*HTR1B* and *HTR2A*). For these analyses, all genotyped SNPs within 5 kb of the gene boundaries were analyzed. Additional details on genotype call rates, evaluation of biological sex and imputation are available in the Supplementary Material^2^.

#### Clinical assessment

PTSD was assessed using the Clinician Administered PTSD Scale for DSM-IV (CAPS-IV; Blake et al., 1993) by doctoral level psychologists with diagnoses determined through a consensus meeting consisting of at least three psychologists. Analyses reported here were based on symptoms present within the past 30 days (current PTSD). Major depression and other psychiatric diagnoses were assessed using the Structured Clinical Interview for DSM-IV (SCID-IV; First et al., 1994). The Boston Assessment of Traumatic Brain Injury-Lifetime (BAT-L; Fortier et al., 2013) was used to assess history of blast and TBI. The BATL-L is a semi-structured interview developed for use with combat veterans that covers self-reported history of head trauma and blast exposure, including classification of each blast exposure event into a distance range. The Combat Experiences Scale of the Deployment Risk and Resiliency Inventory (DRRI; Vogt et al., 2012, 2013) was used to assess combat exposure.

#### Genetic analysis

All variables were screened for outliers. This process identified one multivariate outlier that was >3.0 *SD*s from the sample mean on measures of both DMN connectivity and blast exposure and, on this basis, was dropped from further analysis yielding a final sample of *n* = 134. A hierarchical linear regression model was used for analysis. In the first step of each analysis the eight DMN variables were regressed onto the clinical phenotype (e.g., PTSD severity or diagnosis, depending on the analysis; see below) along with the covariates age, sex, and the first two genetic PCs. The SNPs were evaluated individually in a second block and each phenotype × SNP interaction (with corresponding main effects included in the model) was examined in a third. Both nominal (uncorrected) significance and multiple-testing corrected significance (*p*-corrected, adjusting for analysis of multiple SNPs and DMN components) for main effects and interactions were determined using Monte-Carlo null simulation with 10,000 replicates in which the SNP data were randomly permuted between subjects. This analysis imposed strict multiple-testing control while taking into account the correlations between SNPs and between the eight DMN variables. Analyses of potential confounding variables were examined in *post-hoc* analyses using the same model.

## Results

### Sample characteristics

Descriptive statistics for the primary study variables are listed in Table 2 by current PTSD diagnosis. PTSD cases and controls did not differ significantly in mean age or close-range blast exposure. Participants with PTSD reported significantly more lifetime trauma and were more likely to have a diagnosis of major depression and be taking an anti-depressant medication compared to controls.

### Candidate SNP association analysis

In the first block of the genetic association analysis, the eight DMN variables were regressed onto age, sex, the two genetic PCs and PTSD severity. Results showed a significant main effect of sex on connectivity between the PCC and the left mPFC (β = 0.115, *p* < 0.002) and within the PCC-left MTG circuit (β = 0.095, *p* = 0.026) with women showing greater DMN connectivity in both components compared to men but no PTSD main effect (results for males-only are reported below). When the seven candidate SNPs were entered in the second block of the analysis, results showed a significant main effect of rs130058 (*HTR1B*) on connectivity between the PCC and right angular gyrus with carriers of the A (minor) allele tending to show reduced connectivity within this region compared to others (see Table 3). Analysis of each PTSD severity × SNP interaction in the third block revealed multiple testing-corrected significant interactions (corrected for the seven SNPs and 8 DMN variables) involving rs7997012 (*HTR2A*) and both the right mPFC (β = 0.002, *p*_corrected_ = 0.005) and right MTG components (β = 0.002, *p*_corrected_ = 0.017). We then examined the generalizability of the rs7997012 × PTSD severity effect to a clinical diagnosis of PTSD by repeating the analysis using this one SNP and a dichotomous current PTSD diagnosis variable and found a significant interaction involving the right MTG component that survived multiple testing correction across the 8 DMN components (β = 0.115, *p*_corrected_ = 0.008). Decomposition of the rs7997012 × PTSD severity interaction revealed no significant simple effects, though there was a non-significant trend toward a negative correlation between PTSD severity and right mPFC connectivity (*r* = −0.270, *p* = 0.08 [2-tailed]) among individuals who were homozygous for the G-allele.

**Table 3:** **Top associations (***p***_***corr***_ < 0.10) with DMN components from the 7 candidate SNP analysis**.

**Model parameter**	**DMN component**	**SNP**	**β**	***SE***	***t*-value**	***p*-value**	***p_corr_*^a^**
SNP main effect	Right Angular	rs130058	−0.0608	0.0163	−3.7125	0.0003	0.0154
SNP × PTSD interactions	Right mPFC	rs7997012	0.0019	0.0005	3.9281	0.0001	0.0053
	Right MTG	rs7997012	0.0022	0.0006	3.6123	0.0004	0.0168
	Right Angular	rs7997012	0.0017	0.0005	3.2582	0.0014	0.053
	Left MTG	rs7997012	0.0018	0.0005	3.2014	0.0017	0.0664

a *P-corrected (p_corr_) refers to estimate derived from multiple-testing correction across the 7 SNPs and eight DMN measures examined in this analysis. rs130058 is located in HTR1B, rs7997012 is in HTR2A. Age, sex, and the first two genetic PCs were included as covariates. Angular, Angular Gyrus; mPFC, Medial Prefrontal Cortex; MTG, Middle Temporal Gyrus*.

Given the main effect of sex observed in the first block of the initial analysis, but a female subsample size (*n* = 11) insufficient for meaningful *post-hoc* analysis, we then repeated the original analysis using data from men only and found the same pattern of results with similar multiple-testing corrected effects. We also examined the effects of potential confounding variables by repeating the rs7997012 × PTSD severity analysis using the PCC-right mPFC component as the sole dependent variable with blast exposure, current major depression, antidepressant medication status, and combat exposure included as covariates. None of these covariates contributed significantly to the model and the rs7997012 × PTSD severity interaction term remained significant (*p* = 6 × 10^−5^). Finally, in a separate analysis controlling for age, sex and the two PCs, we examined the association between these seven SNPs and PTSD and found no significant SNP effects.

### *HTR1B* and *HTR2A* gene-wide analysis

**Figure 2:**
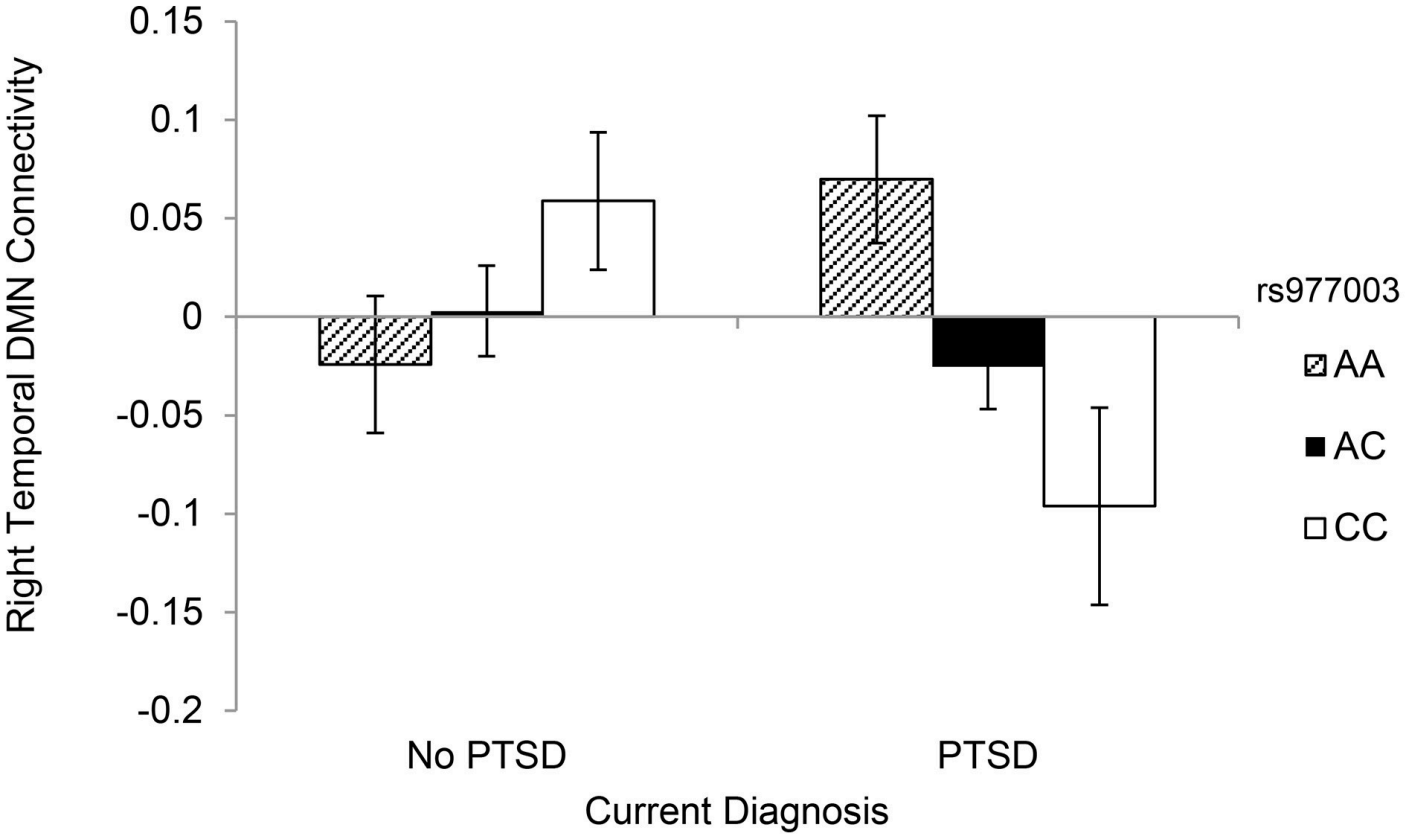
**DMN connectivity within the PCC-right middle temporal gyrus component as a function of rs977003 genotype and current PTSD diagnosis**.

Next, we expanded our examination of the two genes implicated in the candidate SNP analysis to all 99 of the SNPs included within these two regions (11 *HTR1B* SNPs and 88 *HTR2A* SNPs) using the same approach. This analysis revealed two PTSD × SNP interactions that survived correction for multiple testing across the 99 SNPs and 8 DMN components included in the analysis. Specifically, *HTR2A* SNPs rs977003 and rs7322347 moderated the association between PTSD severity and reduced connectivity of the PCC-right MTG component (Table 4; Figure 2). The rs7997012 × PTSD interaction involving the right mPFC that was observed in the candidate SNP analysis fell just short of significant in this analysis (*p*_corrected_ = 0.0503), while the interaction involving the right MTG component fell farther out of range. [The full list of nominally significant associations (i.e., with uncorrected *p* < 0.01) can be found in Table S2].

**Table 4:** **Top associations (***p***_***corr***_ < 0.10) with DMN components from the 99 SNP ***HTR1B*** and ***HTR2A***-wide analysis**.

**Model parameter**	**DMN component**	**SNP**	**β**	***SE***	***t*-value**	***p*-value**	***p_*corr*_*^a^**
SNP × PTSD interactions	Right MTG	rs977003	−0.00253	0.0006	−4.1046	7.23E-05	0.0282
	Right MTG	rs7322347	−0.00253	0.0006	−4.0406	9.21E-05	0.0352
	Right mPFC	rs7997012	0.00193	0.0004	3.9281	0.0001	0.0503
	Right mPFC	rs7322347	−0.00193	0.0004	−3.8892	0.0001	0.0575
	Right mPFC	rs1328684	0.00206	0.0005	3.8631	0.0001	0.0623

a *p_corr_ refers to significance estimate derived from multiple-testing correction across the 11 HTR1B and 88 HTR2A SNPs and eight DMN measures examined in this analysis. Age, sex, and the first two genetic PCs were included as covariates. No SNP main effects were significant. All of the SNPs listed in this table were from HTR2A. mPFC, Medial Prefrontal Cortex; MTG, Middle Temporal Gyrus*.

Because rs977003 and rs7322347 are in high LD (*r*^2^ = 0.97; Reuveni et al., 2016) we chose one of these two SNPs (rs977003) for decomposing the interaction with PTSD involving PCC-right MTG connectivity. First, we examined the correlation between PTSD and DMN connectivity for each genotype separately with age, sex, and the two PCs included as covariates. This showed a significant negative correlation between PTSD severity and DMN connectivity among individuals with two copies of the minor (C) allele only (2 copies: *n* = 25, *r* = −0.542, *p* = 0.010; 1 copy: *n* = 70, *r* = −0.028, *p* = 0.82; 0 copies: *n* = 39, *r* = 0.287, *p* = 0.09). Next, we examined the generalizability of this result to a clinical diagnosis of PTSD by analyzing the effect of genotype in PTSD cases and controls separately. This yielded a significant effect of genotype in cases [*F*_(2, 72)_ = 3.996, *p* = 0.023] but not controls [*F*_(2, 62)_ = 1.748, *p* = 0.183]. Finally, as with the candidate SNP analysis, we then performed a separate analysis examining the association between each of the 99 SNPs and PTSD and found no multiple testing-corrected effects.

#### Major depression, blast exposure, and anti-depressant medication analyses

In light of prior evidence (reviewed earlier) for the influence of close-range blast exposure, anti-depressant medication use and major depression on DMN connectivity, we then examined the specificity of the rs977003 × PTSD interaction by substituting each of these variables, in turn, for PTSD as the interaction term of the model in separate analyses. Neither blast exposure nor anti-depressant medication showed significant main effects or interactions with rs977003 in predicting right MTG connectivity. In the major depression model, the diagnosis × rs977003 interaction was significant (β = −0.1715, *p* = 0.00375) and in the same direction as the PTSD × rs977003 effect. However, when both interaction terms were entered into the same model, the PTSD interaction remained significant (β = −0.1047, *p* = 0.0074) while the depression interaction did not (β = −0.1096, *p* = 0.075).

#### Whole cortex vertex-wise analysis of the rs977003 × PTSD interaction

**Figure 3:**
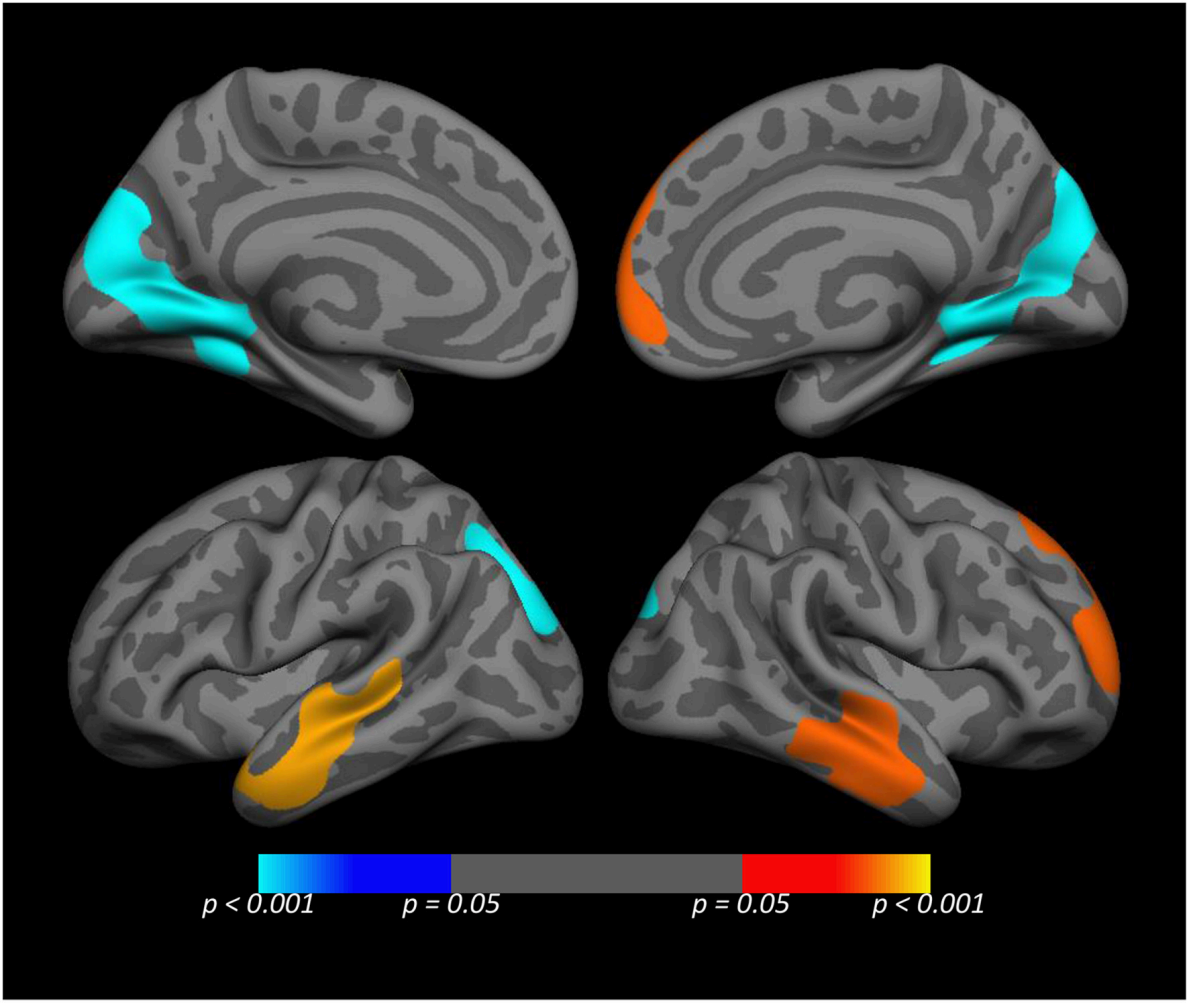
**Medial (top) and lateral (bottom) views of the whole cortex vertex-wise analysis of the rs977003 × PTSD diagnosis interaction**. The cluster-wise significance for all clusters depicted was *p* ≤ 0.0051. The temporal and frontal clusters (red/yellow) correspond to components of the DMN modeled in the primary analysis. The blue cluster in the visual cortex, (which was opposite in direction relative to the temporal and frontal effects) was not detected in the primary analysis because it is not a component of the DMN.

To obtain a more precise localization of the most significant SNP × PTSD interaction, we performed a whole cortex vertex-wise analysis of the most significant SNP rs977003 (Figure 3). This indicated that the locus of the pre-frontal effect fell in portions of the superior frontal gyrus, while the temporal lobe effect was primarily within portions of the middle and inferior temporal gyri. Unexpectedly, this analysis revealed a new cluster in the occipital cortex (involving portions of the cuneus, lingual gyrus and pericalcarine cortex) showing an opposite effect.

## Discussion

This study examined the hypothesis that serotonin receptor gene variants moderate the association between PTSD and DMN connectivity and, to our knowledge, was the largest DMN study of PTSD conducted to date, as well as the first to examine associations between serotonin receptor genes and DMN connectivity in any sample. On the basis of prior evidence for a modulatory role of the serotonergic system on DMN activity, we began by examining candidate SNPs in the *HTR1A, HTR1B, HTR2A*, and *HTR3B* genes that had been implicated previously in quantitative meta-analyses of relevant psychiatric traits. Results of this analysis showed that the anti-depressant medication response-associated *HTR2A* SNP rs7997012 (Niitsu et al., 2013; Lin et al., 2014) interacted significantly with PTSD severity and diagnostic status in predicting reduced connectivity between the PCC and both the right mPFC and right MTG components of the DMN network among PTSD patients but not controls. We also observed a main effect association between the *HTR1B* SNP rs130058 (previously implicated in risk for substance abuse; Cao et al., 2013) and connectivity between the PCC and right angular gyrus component of the network. Based on the locations of these SNPs, we then expanded our analysis to the full array of 99 *HTR1B* and *HTR2A* SNPs. In this analysis, the rs7997012 interaction fell just short of the threshold for multiple testing-corrected significance, while two other *HTR2A* SNPs in high LD with each other (rs977003 and rs7322347) emerged as significant moderators of the association between PTSD and DMN such that individuals with PTSD who were homozygous for the minor allele of these SNPs showed reduced connectivity within the PCC-right MTG component of the DMN.

rs7997012 is the best-known of the 3 SNPs implicated in this study and has been the focus of ~1 dozen studies of anti-depressant medication response (for meta-analyses, see Niitsu et al., 2013; Lin et al., 2014) and it was the top SNP associated with several psychiatric phenotypes in reports from the Sequenced Treatment Alternatives for Depression study (STAR^*^D; Peters et al., 2004; McMahon et al., 2006; Paddock et al., 2007). In that landmark study of 1953 patients with major depressive disorder, investigators genotyped 768 SNPs spanning 68 candidate genes and found that individuals homozygous for the rs7997012 A (minor) allele showed more favorable responses to the SSRI medication citalopram. These results, combined with evidence for this SNP's influence on serotonin transporter binding potential, led Laje et al. (2010) to conclude that individuals homozygous for the rs7997012 A allele likely have lower basal 5-HT transmission, and as a function of this, are more likely than G-allele carriers to derive beneficial effects of 5-HT reuptake inhibition via SSRIs. In our study, G-allele homozygotes with PTSD tended to show reduced DMN connectivity between the PCC and right mPFC, which, like the pharmacologic studies reviewed earlier, points to a negative association between serotonergic tone and DMN connectivity.

Considerably less is known about the two SNPs (rs977003 and rs7322347) that moderated the association between PTSD and DMN connectivity in the *HTR1B* and *HTR2A* gene-wide analysis. These two SNPs are in nearly perfect LD (*r*^2^ = 0.97; 1000 Genomes Project Consortium, 2012) and in moderate LD with rs7997012 (*r*^2^ = 0.60; 1000 Genomes Project Consortium, 2012). rs977003 showed a nominally-significant association with serotonin transporter binding potential in the previously mentioned study by Laje et al. (2010) but has received little additional attention to date. rs7322347, on the other hand, has been linked in prior candidate gene studies to aggression (Banlaki et al., 2015), attention-deficit hyperactivity disorder (Ribases et al., 2009), and suicide attempts (Ben-Efraim et al., 2013). It is an intronic variant known to disrupt a potential miRNA binding site (Banlaki et al., 2015), but its role in gene regulation and receptor function has yet to be studied.

The primary DMN component implicated in this study was connectivity between the PCC and right MTG. Structurally, these regions are inter-connected by white matter fiber bundles (Gong et al., 2009; Bajaj et al., 2013). Functionally, they are subsumed within the broader dorsal-medial-prefrontal cortex DMN subsystem (Andrews-Hanna et al., 2010). This subsystem is preferentially active during stimulus-independent thought (e.g., mind wandering) and when participants are asked to make self-referential judgments about their present situation or mental states (Buckner et al., 2008). The PCC has been shown to be involved in self-monitoring and awareness (Vogt and Laureys, 2005), self-reflection (Johnson et al., 2002), and determining emotional salience (Maddock et al., 2003). The role of the right MTG in the DMN is somewhat less clear; however, reduced resting state connectivity within this region has been reported in prior PTSD studies (Bluhm et al., 2009; Qin et al., 2012). Furthermore, Ludäscher et al. (2010) reported a significant negative correlation between ratings of the intensity of a dissociative experience and BOLD signals in the right MTG (Porcelli et al., 2012) during processing of dissociation-inducing scripts in patients with borderline personality disorder. From another perspective, evidence also suggests that reduced DMN connectivity in PTSD reflects the predominant mobilization of the salience network, which is incompatible with DMN activity and more strongly associated with anxiety (Kumari et al., 2007), the detection of negative stimuli (Muehlhan et al., 2015) and hypervigilance (e.g., Hayes et al., 2012; Sripada et al., 2012).

Finally, to obtain a more precise localization of the SNP × PTSD diagnosis interaction, we performed a whole cortex vertex-wise analysis of the most significant SNP × PTSD interaction (involving rs977003). This showed the locus of the pre-frontal effect to be in portions of the superior frontal gyrus, while the temporal lobe effect was strongest within portions of the middle and inferior temporal gyri. Unexpectedly, this analysis revealed a new cluster in the occipital cortex, showing the opposite effect, which was not found in the original analyses because it is not part of the DMN (specifically, located within portions of the cuneus, lingual gyrus, and pericalcarine cortex). These regions have well-established roles in the processing of visual stimuli, yet in the context of a resting state scan the functional significance of connectivity in this region is not obvious. However, one theoretical process that could potentially account for this finding is *perceptual decoupling*, i.e., the concept that as consciousness becomes focused on internally-generated thoughts, external input loses its influence on attentional processes (Smallwood, 2013). Our finding of an inverse pattern of connectivity between the visual cortex and nodes of the DMN is broadly consistent with this hypothesis, though to our knowledge, unprecedented in the PTSD literature.

These findings should be considered in light of several study limitations including the cross-sectional assessment and ancestrally homogenous and predominantly male sample. The DMN component implicated in our findings was functionally and anatomically distinct from the region where we reported associations with close-range blast exposure in a prior study using the same cohort (i.e., the somatosensory and motor cortices; Robinson et al., 2015). However, the analytic approach and the genotype-confirmed WNH male sample that this study was based on differed substantially from that used in our prior report. We also had no direct measures of serotonergic tone or serotonin receptor binding potential, so inferences about the modulatory effects of serotonin on the DMN are speculative and based on indirect evidence from other studies. Results should also be weighed while considering the unmeasured/unanalyzed aspects of environment, the heterogeneity of the disorder, and potential gene × gene interactions underlying PTSD clinical presentations that we were unable to model given the size of our sample.

In conclusion, research on the genetics and neurobiology of PTSD has historically focused largely on identifying risk factors that give rise to the syndrome through their interaction with trauma exposure. In this study, we focused instead on identifying genetic variants that modify the association between a hypothesized endophenotype of PTSD and the clinical diagnosis with the hope that doing so will advance our understanding of sources of individual variability in the association between the putative biomarker and the phenotype. Results add to a growing body of research suggesting a modulatory influence of the 5-HT_2A_ receptor on DMN connectivity. More broadly, they contribute to an extensive literature on the role of the 5-HT_2A_ receptor in neural mechanisms of cognitive control and the modulation of anxiety (Aznar and Klein, 2013). Findings also point to the potential value of the DMN paradigm in future studies of SSRI treatment response and efforts toward the development of genetically-informed methods of treatment matching.

## Author contributions

MM and ES designed the study, analyzed the data, interpreted the results, and wrote up the findings. MR, NS, EW, JH, ML, SS, AS, WM, and RM helped interpret the results, revised the work, and approved the version to be published. All authors agree to be accountable for all aspects of the work in ensuring that questions related to the accuracy or integrity of any part of the work are appropriately investigated and resolved.

### Conflict of interest statement

ES, MR, NS, EW, JH, ML, SS, AS, WM, and RM declare that the research was conducted in the absence of any commercial or financial relationships that could be construed as a potential conflict of interest. MM owns stock in Illumina, Inc.

## Footnotes

^1^*5-HTTLPR* is a 43-base pair insertion/deletion length polymorphism. There are no individual SNPs or SNP haplotypes that offer perfect proxies for the traditional *5-HTTLPR* genotypes so we could not examine it in this study.

^2^The US Department of Veterans Affairs (VA) currently prohibits the release of genomic information from veterans outside of the VA. Due to this, we are unable to comply with Frontiers' data sharing policy at this time though summary statistics are available for meta-analysis and collaboration on secondary/replication analysis with the corresponding author is welcome.
