# Supplementary material for: 5-HT2A Gene Variants Moderate the Association between PTSD and Reduced Default Mode Network Connectivity
Source: Front Neurosci. 2016 Jun 28;10:299. doi: 10.3389/fnins.2016.00299 (PMC4923242; doi:10.3389/fnins.2016.00299)
Supplement: Supplementary file 1 [file DataSheet1.DOCX]

***Supplementary Material***

**5-HT_2A_ Gene Variants Moderate the Association between PTSD and Reduced Default Mode Network Connectivity**

**Mark W. Miller^1,2*^, Emily Sperbeck^2^, Meghan E. Robinson^3-5^, Naomi Sadeh^1,2^, Erika J. Wolf^1,2^, Jasmeet P. Hayes^1,2^, Mark Logue^1,2,6,7^, Steven A. Schichman^8^, Angie Stone^8^, William Milberg^9-10^, and Regina McGlinchey^9-10^**

***Correspondence:** Mark W. Miller: mark.miller5@va.gov

**Genotyping: Laboratory Procedures**

DNA was isolated from peripheral blood samples on a Qiagen AutoPure instrument with Qiagen reagents; concentrations were normalized using the Quant-iT™ PicoGreen dsDNA fluorescent assay (Invitrogen). DNA quality and quantity were ascertained by the TaqMan® RNase P Detection assay (Applied Biosystems Assay, Life Technologies, Carlsbad, CA) with fluorescence detection on a 7900 Fast Real Time PCR System (Applied Biosystems, Life Technologies, Carlsbad, CA) according to the manufacturer's protocol. DNA samples were whole-genome amplified, fragmented, precipitated and resuspended prior to hybridization on Illumina HumanOmni2.5-8 beadchips for 20 hours at 48⁰C according to the manufacturer’s protocol (Illumina, San Diego, CA). After hybridization, a single-base extension followed by a multi-layered staining process was performed. Beadchips were imaged using the Illumina iScan System and analyzed with Illumina GenomeStudio v2011.1 software containing Genotyping v1.9.4 module. A GenomeStudio project was created with a custom genotyping cluster file, and call rates were >0.994 for all samples. Technical replicates had genotyping reproducibility error rates <0.0005 prior to SNP data cleaning.

**Genotyping: Statistical Procedures**

SNP data cleaning and manipulation was performed using PLINK8. X-chromosome genotypes were concordant with self-report sex in all cases. IBD analysis was used to check for cryptic relatedness in the sample. Genetic data for all 218 subjects with self-reported white non-Hispanic (WNH) were submitted to the following pipeline though DMN data was only available for a subsample of 135 of these subjects. Concordance between self-reported and genetically predicted ancestry was investigated using principal components (PC) analysis as implemented in EIGENSTRAT9, based on the genotypes of 100,000 common SNPs. First, PC analysis was performed together with 1,000 Genomes reference data for the EUR, AFR, ASN, and AMR samples. Of the 218 subjects, 4 were more than 6 SD away from the WNH group mean on the generated PC and were removed from the analysis, leaving a sample of 214. Next, PCs were calculated for use as analysis covariates using the remaining 214 subjects. None of the subjects were outliers (>6SD) in this PC analysis. Then, genotypes for SNPs not covered on the 2.5^-8^ array were generated using Impute2 (Howie et al., 2009) and 1000 Genomes reference data (1000 Genomes Project Consortium, 2012). Prior to imputation, all C/G and A/T SNPs, SNPs with missing rates > 1%, and SNPs with Hardy-Weinberg equilibrium (HWE) test p-values <10^-6^ were removed. Imputation was performed using 1000 Genomes phase 1 integrated haplotypes (June 2014), based on only the haplotypes for the 379 EUR reference-panel subjects in order to reduce computational cost.

**References**

1000 Genomes Project Consortium. (2012). An integrated map of genetic variation from 1,092 human genomes. *Nature*, 491, 56-65.

Howie B. N., Donnelly P., and Marchini J. (2009). A flexible and accurate genotype imputation method for the next generation of genome-wide association studies. *PLoS Genet.*, 5, e1000529.

Table S1. Correlations between DMN components.

| **DMN Region** | **1** | **2** | **3** | **4** | **5** | **6** | **7** |
| --- | --- | --- | --- | --- | --- | --- | --- |
| **1. Left mPFC** | - |  |  |  |  |  |  |
| **2. Left Cingulate** | .283** | - |  |  |  |  |  |
| **3. Left Angular** | .575** | .349** | - |  |  |  |  |
| **4. Left MTG** | .616** | .141 | .528** | - |  |  |  |
| **5. Right mPFC** | .760** | .334** | .420** | .607** | - |  |  |
| **6. Right Cingulate** | -.028 | .698** | .135 | .027 | .306** | - |  |
| **7. Right Angular** | .340** | .280** | .526** | .456** | .594** | .375** | - |
| **8. Right MTG** | .463** | .223* | .347** | .761** | .629** | .240** | .494** |

Cingulate = Isthmus of the Cingulate; Angular = Angular Gyrus; mPFC = Medial Prefrontal Cortex; MTG = Middle Temporal Gyrus

**p* < .05, ***p* < .01, ****p* < .001

Table S2. Nominally significant associations (*p* < .01 uncorrected) with DMN components from the 99 SNP *HTR1B* & *HTR2A*-wide analysis.

| **Model Parameter** | **DMN component** | **SNP** | ***β*** | **SE** | **t-value** | **p-value** | ***p_corr_*^a^** |
| --- | --- | --- | --- | --- | --- | --- | --- |
| **SNP x PTSD interactions** | **Left MTG** | rs7322347 | -0.0021 | 0.0006 | -3.6887 | 0.0003 | 0.1059 |
|  | **Right mPFC** | rs977003 | -0.0018 | 0.0005 | -3.6862 | 0.0003 | 0.1068 |
|  | **Right mPFC** | rs1328677 | 0.0021 | 0.0006 | 3.6531 | 0.0004 | 0.1193 |
|  | **Right MTG** | rs7997012 | 0.0023 | 0.0006 | 3.6124 | 0.0004 | 0.1336 |
|  | **Right mPFC** | rs10507544 | 0.0018 | 0.0005 | 3.5643 | 0.0005 | 0.1559 |
|  | **Left MTG** | rs977003 | -0.0020 | 0.0006 | -2.5027 | 0.0006 | 0.1841 |
|  | **Right mPFC** | rs9567732 | 0.0018 | 0.0005 | 3.4687 | 0.0007 | 0.2043 |
|  | **Right MTG** | rs1923885 | -0.0022 | 0.0006 | -3.4519 | 0.0008 | 0.2156 |
|  | **Right mPFC** | rs9567737 | 0.0017 | 0.0005 | 3.4449 | 0.0008 | 0.2188 |
|  | **Right MTG** | rs6561332 | -0.0022 | 0.0007 | -3.4124 | 0.0009 | 0.2406 |
|  | **Right MTG** | rs7330636 | -0.0022 | 0.0007 | -3.3565 | 0.001 | 0.2748 |
|  | **Right mPFC** | rs6561332 | -0.0017 | 0.0005 | -3.3429 | 0.0011 | 0.2834 |
|  | **Right MTG** | rs9567731 | 0.0023 | 0.0007 | 3.2813 | 0.0013 | 0.3287 |
|  | **Right Angular** | rs10507544 | 0.0017 | 0.0005 | 3.2605 | 0.0014 | 0.3456 |
|  | **Right Angular** | rs7997012 | 0.0018 | 0.0005 | 3.2583 | 0.0014 | 0.3470 |
|  | **Right MTG** | rs10507544 | 0.0021 | 0.0006 | 3.2341 | 0.0016 | 0.3672 |
|  | **Left MTG** | rs7997012 | 0.0018 | 0.0006 | 3.2015 | 0.0017 | 0.3954 |
|  | **Right mPFC** | rs1923885 | -0.0016 | 0.0005 | -3.1354 | 0.0021 | 0.4507 |
|  | **Right Angular** | rs7322347 | -0.0017 | 0.0006 | -3.1149 | 0.0023 | 0.4688 |
|  | **Left Cingulate** | rs9567737 | 0.0010 | 0.0003 | 3.0798 | 0.0025 | 0.5030 |
|  | **Right Angular** | rs1328677 | 0.0019 | 0.0006 | 2.9922 | 0.0033 | 0.5928 |
|  | **Right Angular** | rs977003 | -0.0016 | 0.0005 | -2.9606 | 0.0037 | 0.6237 |
|  | **Right mPFC** | rs7330636 | -0.0016 | 0.0005 | -2.9603 | 0.0037 | 0.6249 |
|  | **Right MTG** | rs9567732 | 0.0019 | 0.0007 | 2.9544 | 0.0037 | 0.6306 |
|  | **Left mPFC** | rs7997012 | 0.0014 | 0.0005 | 2.9368 | 0.0039 | 0.6493 |
|  | **Right Angular** | rs9567737 | 0.0016 | 0.0006 | 2.9240 | 0.0041 | 0.6601 |
|  | **Right Angular** | rs9567732 | 0.0016 | 0.0006 | 2.9092 | 0.0043 | 0.6740 |
|  | **Right Angular** | rs6313 | -0.0014 | 0.0005 | -2.8063 | 0.0058 | 0.7719 |
|  | **Left MTG** | rs6561332 | -0.0017 | 0.0006 | -2.8033 | 0.0059 | 0.7737 |
|  | **Right MTG** | rs9567737 | 0.0018 | 0.0006 | 2.7928 | 0.006 | 0.7809 |
|  | **Right MTG** | rs56005991 | -0.0021 | 0.0008 | -2.7398 | 0.007 | 0.8222 |
|  | **Right mPFC** | rs9567731 | 0.0015 | 0.0005 | 2.7375 | 0.0071 | 0.8234 |
|  | **Right mPFC** | rs1928042 | 0.0017 | 0.0006 | 2.7232 | 0.0074 | 0.8331 |
|  | **Right Angular** | rs732821 | -0.0014 | 0.0005 | -2.7088 | 0.0077 | 0.8414 |
|  | **Right Cingulate** | rs9567737 | 0.0009 | 0.0003 | 2.7048 | 0.0078 | 0.8438 |
|  | **Right Angular** | rs6561332 | -0.0016 | 0.0006 | -2.6958 | 0.008 | 0.8503 |
|  | **Right mPFC** | rs1328685 | 0.0019 | 0.0007 | 2.6955 | 0.008 | 0.8505 |
|  | **Left MTG** | rs10507544 | 0.0016 | 0.0006 | 2.6945 | 0.008 | 0.8519 |
|  | **Right Angular** | rs6311 | -0.0014 | 0.0005 | -2.6670 | 0.0087 | 0.8695 |
|  | **Left mPFC** | rs10507544 | 0.0013 | 0.0005 | 2.6614 | 0.0088 | 0.8724 |
|  | **Left mPFC** | rs1328684 | 0.0014 | 0.0005 | 2.6553 | 0.0089 | 0.8762 |
| **SNP Main Effects** | **Right Angular** | rs130058 | -0.0608 | 0.0164 | -3.7126 | 0.0003 | 0.1173 |
|  | **Left Cingulate** | rs61948307 | 0.0670 | 0.0189 | 3.5505 | 0.0005 | 0.1859 |
|  | **Right Angular** | rs9567731 | -0.0527 | 0.0164 | -3.2135 | 0.0017 | 0.4227 |
|  | **Left MTG** | rs7984966 | -0.0517 | 0.0177 | -2.9169 | 0.0042 | 0.6948 |
|  | **Left MTG** | rs6561332 | -0.0482 | 0.0167 | -2.8791 | 0.0047 | 0.7270 |
|  | **Left MTG** | rs9567737 | 0.0454 | 0.0166 | 2.7288 | 0.0073 | 0.8515 |

^a^*p_corr_* refers to significance estimate derived from multiple-testing correction across the 11 *HTR1B* and 88 *HTR2A* SNPs and eight DMN variables examined in this analysis. Age, sex, and the first two genetic PCs were included as covariates. Cingulate = Isthmus

of the Cingulate; Angular = Angular Gyrus; mPFC = Medial Prefrontal Cortex; MTG = Middle Temporal Gyrus
